# The Deformation Behavior and Bending Emissions of ZnO Microwire Affected by Deformation-Induced Defects and Thermal Tunneling Effect

**DOI:** 10.3390/s21175887

**Published:** 2021-09-01

**Authors:** Linlin Shi, Hong Wang, Xiaohui Ma, Yunpeng Wang, Fei Wang, Dongxu Zhao, Dezhen Shen

**Affiliations:** 1 State Key Laboratory of High Power Semiconductor Laser, Changchun University of Science and Technology, No. 7186 Wei-Xing Road, Changchun 130022, China; 18102475359@163.com (H.W.); mxh@cust.edu.cn (X.M.); 2 State Key Laboratory of Luminescence and Applications, Changchun Institute of Optics, Fine Mechanics and Physics, Chinese Academy of Sciences, No. 3888 Dongnanhu Road, Changchun 130033, China; wangyunpeng@ciomp.ac.cn (Y.W.); wangf@ciomp.ac.cn (F.W.); zhaodx@ciomp.ac.cn (D.Z.); shendz@ciomp.ac.cn (D.S.)

**Keywords:** microwires, deformation behavior, electroluminescence, bending emission

## Abstract

The realization of electrically pumped emitters at micro and nanoscale, especially with flexibility or special shapes is still a goal for prospective fundamental research and application. Herein, zinc oxide (ZnO) microwires were produced to investigate the luminescent properties affected by stress. To exploit the initial stress, room temperature in situ elastic bending stress was applied on the microwires by squeezing between the two approaching electrodes. A novel unrecoverable deformation phenomenon was observed by applying a large enough voltage, resulting in the formation of additional defects at bent regions. The electrical characteristics of the microwire changed with the applied bending deformation due to the introduction of defects by stress. When the injection current exceeded certain values, bright emission was observed at bent regions, ZnO microwires showed illumination at the bent region priority to straight region. The bent emission can be attributed to the effect of thermal tunneling electroluminescence appeared primarily at bent regions. The physical mechanism of the observed thermoluminescence phenomena was analyzed using theoretical simulations. The realization of electrically induced deformation and the related bending emissions in single microwires shows the possibility to fabricate special-shaped light sources and offer a method to develop photoelectronic devices.

## 1. Introduction

With the development of manufacturing technology, nano/microstructures have realized their potential in functional optoelectronic devices such as light-emitting devices, diode lasers, and photodetectors [1,2,3,4,5]. One-dimensional micro/nanostructures, such as nanowires (NWs), nanobelts (NBs), and microwires (MWs), can be employed to expand immense versatility and practicality of modulating the electronic and photonic propagation behavior, as well as optical properties, which are considered to be the effective ways to fabricate multifunctional nanodevices. ZnO micro/nanowires have an instinctive bend ductility and highly efficient thermoelectricity, therefore it is crucial to explore the ZnO micro/nanowires for various applications with shape plasticity as well as application compatibility [6,7,8,9,10]. These micro/nanowires have also attracted a lot of interest owing to their unique semiconducting, photoelectric, and piezoelectric properties for plenty of applications in piezo-optoelectronics, such as nanogenerators [11,12,13,14,15], piezoelectric devices, etc., [16,17,18,19,20,21,22,23]. Most of the previous studies have reported that the crystal and electronic structure of the semiconductor nanowires are sensitive to the applied mechanical strain [24]. Electrical and optoelectrical properties of the nanowires are improved by the elastic strain engineering, which is based on the localized and inhomogeneous elastic strain accompanied by deformation-induced high strain-gradient [25,26]. In the previous reports, the semiconductor parameters of deformed microwires, including resistivity, carrier concentration, and mobility, were extracted from the experimental and theoretical analysis [27,28,29]. However, the effect of mechanical bending deformation of ZnO NWs on their electroluminescent behavior is not much explored [30]. Though the deformation-induced electrical and optical effects are widely studied but the reports on electroluminescence properties are rather minimal [31]. As previously reported that, incandescent-type light sources have been realized on account of individual ZnO microwires [32,33], and the dominant lighting wavelengths can be tuned from visible to near-infrared due to the intentional adjusting the defect level or construction p-n junction [34,35,36]. Therefore, electrically pumped individual ZnO microwires with flexibility and the deformation-induced luminescent properties are still needed to be further studied that are of great significance to explore potential application.

Hence, in this paper, ZnO microwires are produced and bent to the arc shape to induce recoverable deformation to investigate the electrical and electroluminescence properties. The deformation behavior of the ZnO microwires under the continuous external electric current is explored. The morphology and photoluminescence measurements of the ZnO microwires are characterized to investigate the internal lattice changes after deformation. The phenomenon of preferential light emission in deformation area is observed. The finite element analysis method is used for the analysis of thermal potential field distribution along the microwires to discuss the electroluminescence properties.

## 2. Materials and Methods

ZnO microwires were produced by a typical chemical vapor deposition (CVD) technique. Two precursors, namely ZnO powders (Sigma-Aldrich, St. Louis, MO, USA, purity 99.999%) and graphite powders (Sigma-Aldrich, St. Louis, MO, USA, <20 μm), were mixed thoroughly with a weight ratio of 1:1 and were kept in an alumina boat. A 100-nm ZnO film was deposited on the Si substrate by the magnetron sputtering method, and then the substrate was loaded above the ZnO powders. During the synthesis process, the reactant ZnO source was loaded into the furnace with a constant flow of 100 sccm argon (99.99%) as the carrier gas, and then heated to 1100 °C. After maintaining the source temperature for 40 min, the furnace was turned off and cooled down to room temperature. After the synthesis was completed, ultralong ZnO microwires with an average length of about 10 mm were observed (visual observation) on the substrate.

The experiments were carried out by fixing a microwire on an insulating substrate (Si/SiO_2_ substrate and glass substrate). The microwire was bent into a curved configuration on the probe station and was kept under an optical microscope equipped with a CCD camera. Indium paste was used to fix the microwire and was used as the cathode. In the flexible bending experiments, the microwires were attached to polydimethylsiloxane (PDMS, Dow Corning, Midland, MI, USA, SYLGARD 184) substrate. The microwires were first put on top of the polycarbonate filter, which were in close contact by the vacuum suction force. PDMS were used to cover the microwires, after 2 h drying (60 °C), a flexible membrane with 1 mm thickness was obtained. A conductive tape was used to fix the microwire and to connect the cathode with it. In this design, there was no transverse bending or twist on the wire ensuring the stability of the microwire. Straight microwires were prepared on the insulating substrate without any stress.

The morphology of the ZnO microwires was characterized by a field-emission scanning electron microscope (FE-SEM) (model:Hitachi S-4800, Tokyo, Japan) equipped with an X-ray energy dispersive spectrometer (EDX) and high-resolution transmission electron microscope (HRTEM, Hitachi, Tokyo, Japan). Photoluminescence (PL) measurement was carried out with a JY-630 micro-Raman spectrometer (with a resolution of 0.1 nm, HORIBA Jobin Yvon, Palaiseau, France) employing the 325 nm line of a He-Cd laser as the excitation source. Temperature-dependent test was performed at liquid nitrogen temperature from 89 K to room temperature. The current-voltage (I-V) characteristics of microwires were measured using a Keithley 2611A (Keithley Instruments, Solon, OH, USA) measurement system. The EL emissions were collected using a Hitachi F4500 spectrometer (Hitachi, Tokyo, Japan). Optical microscope images and EL emissions images were recorded using an optical microscope. The thermal analysis of the microwire was simulated by using finite element analysis software ANSYS15.0 (Ansys, Canonsburg, PA, USA).

## 3. Results

### 3.1. Deformation Behavior

**Figure 1:**
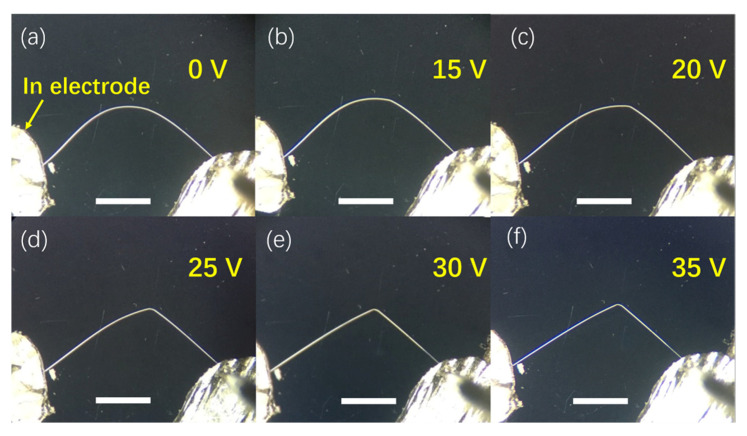
Optical images of the bending ZnO microwire with increasing the applied voltages (from 0 V to 35 V, in (**a**–**f**) respectively). (scale bar: 3 mm).

Constant current was applied on the ZnO microwire by using probes with indium paste, the microwire was bent to an arc shape to acquire internal mechanical stress. The optical image of the bending microwire (under the applied voltage of 0–50 V) is shown in Figure 1. Figure 1a–f displays five sequential images of the deformation process of an individual bent ZnO microwire. From the images, it can be seen that the deformation continuously increases with the increasing applied voltage. However, during the process, the microwire experienced a permanent change in morphology and did not recover its original shape under the applied high voltage. When the voltage exceeded 20 V, the deformation became stable gradually.

**Figure 2:**
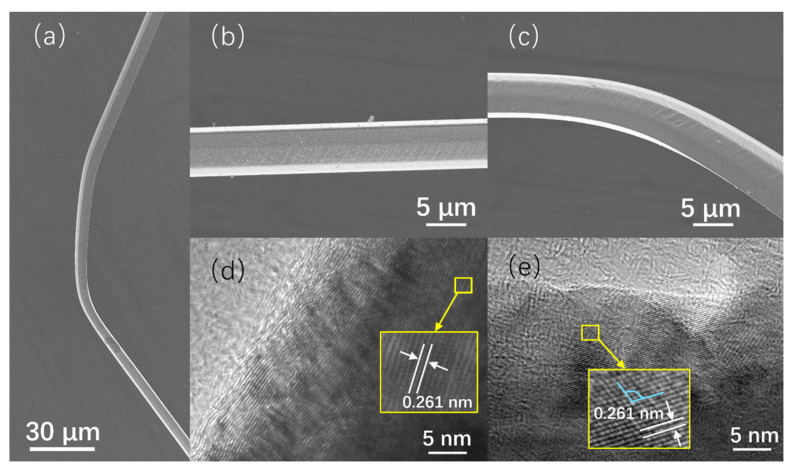
SEM image of the ZnO microwire after bending. (**a**) SEM image of whole bent regions. (**b**,**c**) SEM images of the unbent and bent regions of the ZnO microwire, respectively. (**d**,**e**) The corresponding SAED patterns of the unbent and bent regions of the ZnO microwire.

The straight and the bent region of the ZnO microwire with a cross-section of about 8 µm was characterized by FESEM and HRTEM, and the results are shown in Figure 2. TEM image of the straight microwire shows that the crystal lattice is intact (Figure 2d). In the bending region of the microwire, rippled distortion can be seen from the HRTEM image of the deformed area (Figure 2e). In contrast, the deformed zone reveals several dislocations and misorientations compared to undeformed zone. A huge number of dislocations in the bent wire is mainly due to severe deformation. The bending angle consists of two bigger angles (Figure 2a). The interspacing between two ZnO planes of the bent wire is determined to be 0.261 nm, which matches well with the interspacing of the unbent region. From the electron diffraction spectrum (inset figure), it is seen that the rippled distortion regions have single-crystal characteristics. Therefore, the microwires under the applied voltage have undergone not only a change in shape, but also a change in crystal lattice, which may have significant effects on the electrical transport properties of the microwire at room temperature.

### 3.2. Electrical Behavior

**Figure 3:**
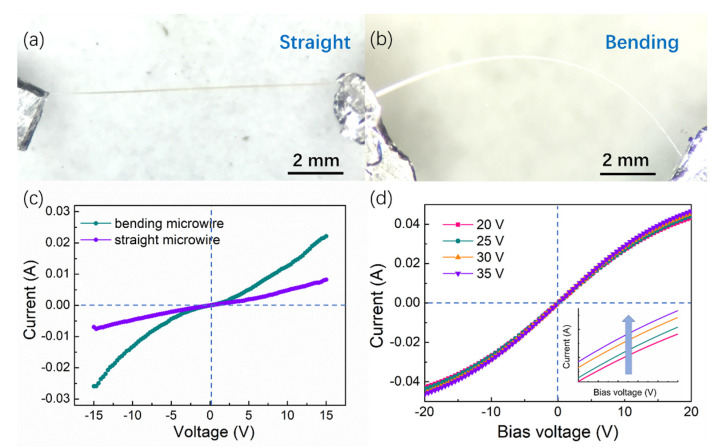
(**a**,**b**) Optical images of the straight and bending microwire under insulating substrate to test I-V characteristics. (**c**) I-V characteristics of the straight microwire and the bending microwire. (**d**) I-V characteristics of bending microwire under different applied voltage.

To determine the electrical conductivity of microwires after bending, current-voltage (I-V) characteristics of the bending and straight microwires are depicted in Figure 3. The I-V curve is nearly linear when the microwire is straight (Figure 3a), which indicate no applied stress on the microwire. The linear I-V characteristic of the nanowire indicates that good ohmic contacts have been achieved. As the microwire was bent to a circle (Figure 3b), the I-V curves obtained at 15 V become nonlinear, which is shown in Figure 3c. Under the applied mechanical bending, the deformation that occurs is recoverable in nature. Hence, the change in conductivity is obvious and it can also return to its original state. When the voltage exceeds 30 V, the deformation becomes unrecoverable, and the supporting I-V curves acquired under 20 V to 50 V are shown in Figure 3d. With the increasing applied voltage, the degree of deformation becomes much larger, and the nonlinearity of the I-V curve gradually increases with the increasing bending deformation, which indicates that the conductivity has also improved after deformation. Therefore, it is demonstrated that bending deformation has an impact on the electrical conductivity of microwires. This can be described by the change in electrons, which occurred due to the deformation-induced strain. However, this unrecoverable deformation is the resultant of the applied voltage and is slightly changed when compared to the recoverable deformation as shown in Figure 3d. It can be concluded from the I-V curves that the conductivity is affected by the degree of deformation. The strong strain gradient developed due to the bending deformation has a significant impact on the electrical properties of the ZnO microwires. Such effects are attributed to the enhancement of carrier mobility induced by the applied compressive stress [37,38].

**Figure 4:**
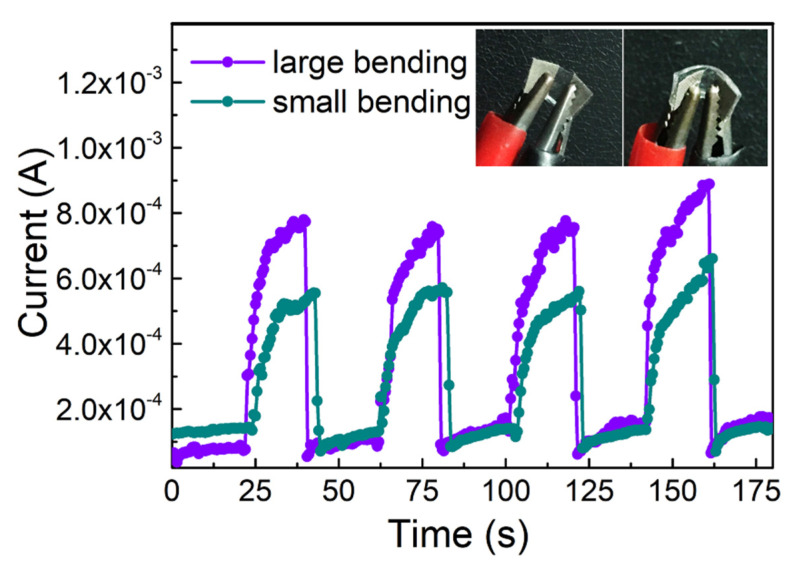
Continuous bending and releasing the stress of ZnO microwires attached to the flexible PDMS substrate. Inset: the images of flexible devices with different bending curvature.

For further demonstrating the relationship between the degree of deformation and conductivity, the I-V characteristics of the microwire fixed by flexible PDMS substrate are displayed in Figure 4, the image of the device is shown in the inset. The external stress on the microwires was applied by bending the PDMS substrate. During bending and releasing the stress, the current curves were recorded consecutively, which are shown in Figure 4. It is displayed that the current remarkably increases with the bending of the microwire under 20 V bias. When the stress was released, the current passing characteristics through the wire were also gradually recovered and reached the initial state, when there was no external stress on the microwire. Though the conductivity of the microwires recovered immediately when the strain was released, the rising current increased slowly. The slow rising process during the experiments might be due to the generation of defects. The electrical conductivity increased by 60% of its original value after bending and then immediately recovered. Moreover, with the increase in the bending curvature, the current ratio became evident. The possible reason for the changes in the deformation-induced transport behavior may be the bending-induced defect formation. The changes in the I-V characteristics indicate the accumulation of charge carriers along the microwire due to the bending deformation.

### 3.3. Photoluminescence Measurements

**Figure 5:**
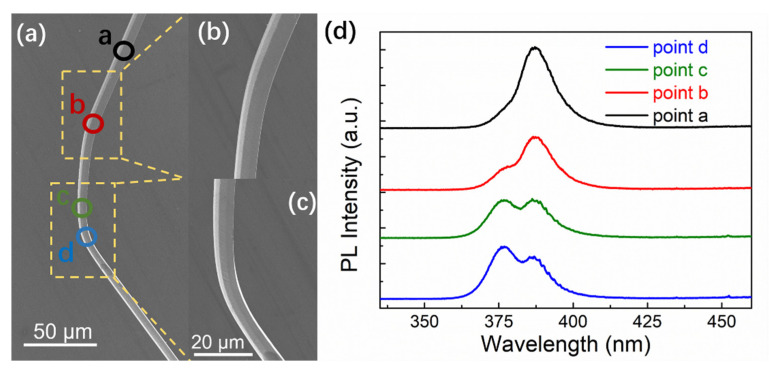
Room temperature PL measurements of different bending regions of ZnO microwire (**d**) and the corresponding focusing areas (**a**–**c**).

In order to investigate the deformation-induced defect formation, photoluminescence (PL) measurements were carried out on the unrecoverable bent ZnO microwires to illustrate the effect of bending strain on the bend structure of the microwires. Figure 5 displays the room temperature PL spectrum of the ZnO microwire in the straight area and bending areas. Local PL measurements were characterized along the axial direction in the bent microwires. The focused areas marked by colored circles are displayed in Figure 5a. The spectra of the straight area (point a) present a dominant emission at 376 nm (3.29 eV), which is related to the near band emission (NBE) at room temperature. The strong NBE emission suggests that the microwire has high crystalline quality. However, when the exciting light moves gradually to the bending deformation position (from point b to d), a new peak of higher energy appears, which can be the resultant of the coupling of energy states by exciton diffusion in the bending ZnO microwire. The states in straight and bending areas are coupled by the exciton diffusion effect in bent ZnO nanowires. the increase of exciton in bending areas result in a recombination in higher energy zone, coinciding with a new peak at high energy side [39]. The variation resulted from the coupling change of electronic energy states in the tensile and compressive parts through exciton diffusion.

**Figure 6:**
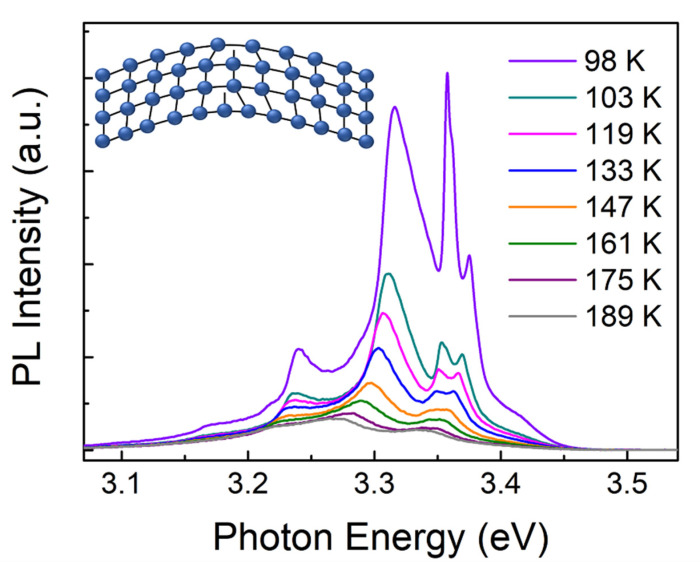
Temperature dependent PL measurements in bending area, inset: the schematic diagram of emerging dangling bounds defect.

Moreover, from the low temperature PL measurements in Figure 6, the new emerging peak of higher energy can also be ascribed to the broken crystal symmetry by the inhomogeneous strain which developed from the change in the lattice. This lattice change gives rise to defects in ZnO microwires. The existing defect state is ascribed to the emerging dangling bounds located at the surface of the bent microwires developed due to the unrecoverable inelastic bending deformation, the schematic diagram is displayed in the inset of Figure 6 [28]. These dangling bounds generate defects such as vacancies and interstitial atoms. It is reported that the crystal imperfections give rise to additional intermediate states, which enhance the exciton-phonon coupling strength in ZnO microwires. The strong emission peak located around 3.37 eV is ascribed to the increase in the recombination of free exciton (FX) emission, which can be related to the modification of surface hole and electron trapping sites located near the surface of the microwire [29]. With higher defect densities increase, the relative intensity of the FX line increases.

### 3.4. Electroluminescence Performance

**Figure 7:**
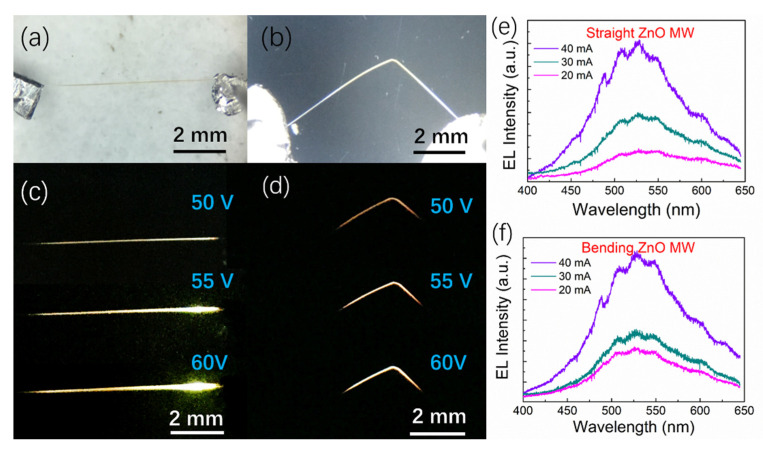
Electroluminescence performance of ZnO microwire. (**a**,**b**) Optical images of straight and bent ZnO microwires, (**c**,**d**) the corresponding emission images of straight and bent ZnO microwires captured by CCD camera, (**e**,**f**) electroluminescence spectrum of straight and bent ZnO microwires.

Electrically driven light emission based on straight and bending microwires were constructed, and the results are presented in Figure 7. Electroluminescence (EL) emission characteristics of a single ZnO microwire using an incandescent-type light source were observed by applying bias on both ends of the bare ZnO microwire. The dominant emission peaks centered at 520 nm are shown in Figure 7e,f, which is accompanied by the defect band generated from vacancies and interstitial atoms. The bright and visible emissions can be directly recorded using optical microscopic CCD for single unbent and unrecoverable bending deformation, as shown in Figure 7c,d. With injection voltage continuing to increase, the brightness and emission regions of microwire-based emitter increase, and the dominant emission wavelength shows no shift. In addition, the light occupies the entire area of the unbent microwire that is observed from the luminous image. However, in the bending deformation of the ZnO microwire, the emission light tends to concentrate on the bent regions. This may be because of the relatively large number of defects concentrated in the bent regions, the external voltage exceeds exciton threshold in bending area, which dominated the emission and is relatively low compared with the unbent regions.

## 4. Discussion

The possible mechanisms for the electroluminescence properties are related to two possibilities. One is the surface defect effect; in this case, the electrons and holes injected from electrodes tend to undergo recombination at the surface of microwires, and the surface defect of ZnO microwires becomes more distinct and collective under bending deformation. The previous reports demonstrate that the emission corresponds to the oxygen vacancy (V_O_) defects of ZnO, the defect creating a thin depletion layer near the surface. The V_O_ defect is the most widely accepted mechanism for the visible emission of ZnO. The origin of the emitting light can be attributed to the electron transition from the defect center to the valence band edge, and the emission light concentrated at bent area is largely due to the accumulation of defects.

**Figure 8:**
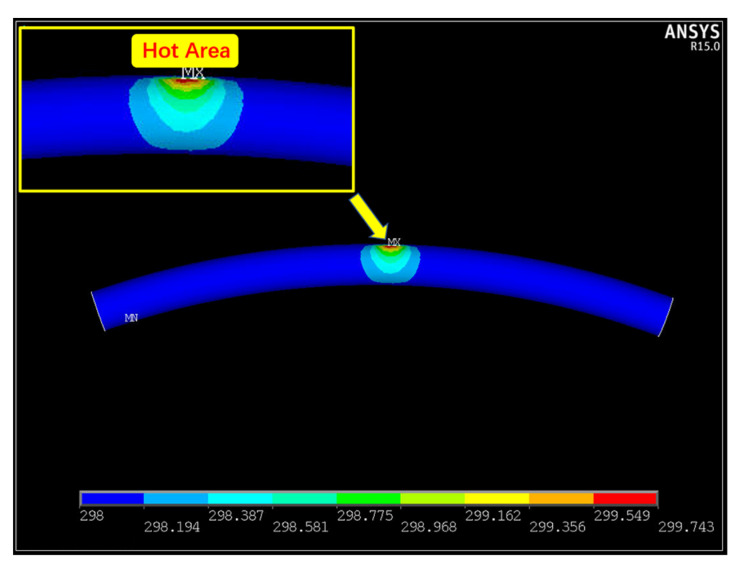
Simulation of temperature distribution along the bending areas of microwire.

The other possibility is the thermal light emission due to Joule heating during the process, which can be defined as the thermal tunneling effect of nanostructures [40]. The emission and current response to bending deformation can be explained by the thermal properties of bending ZnO microwires [41,42]. The thermal potential field distribution along the microwire was simulated by using finite element analysis software ANSYS as exhibited in Figure 8, here the simulation of microwire was established under the condition of equilibration at a constant temperature. The temperature distribution along the microwire illustrates that the highest temperature region is linearly distributed and mainly concentrated on the bending areas, coinciding with the location of defect regions that could influence the electronic states of ZnO [43,44,45,46]. A moderate reduction in the thermal conductivity is demonstrated in the bending ZnO microwires. Furthermore, it shows that the defects tend to concentrate on the compressive and tensive zones of the bent microwire. The electrons under a high bias voltage have sufficient energy to scatter inelastically and then tunnel through the depletion layer to excite the defect states. Furthermore, the deformation of the ZnO microwire in the bending area is another reflection of the thermal tunneling electroluminescence. The defects dominated in the bent area can produce more heat than the straight area, which can also explain the electrical deformation behavior of ZnO microwires.

## 5. Conclusions

A remarkable thermoelectric deformation in the mechanically strained ZnO microwires is reported in this work. Highly crystallized ZnO microwires with several millimeters long were successfully synthesized via the CVD process. Curved ZnO microwire with internal stress present unrecoverable deformation, the deformation continuously increases with the increasing applied voltage. Internal lattice structure of the deformation regions were characterized, the dislocations of the severe deformation region demonstrated the additional defects compared with the undeformed zone acquired from the optical and electrical properties. The changes in conductivity from the I–V characteristics and the new emerging emission peak from PL measurements both gives rise to defects in ZnO microwires. By applying bias onto the microwires, electroluminescence phenomena in bent region priority to straight region was observed. The uncoverable bending emission may be attributed to the involved defects and more defects are concentrated on the bent area. Thermoelectrical thermal tunneling electroluminescence may be a possible explanation for both electrical deformation behavior and the electroluminescence behavior of the ZnO MWs due to the accumulation of heat at the bent area of the ZnO microwire. We believe that the realization of bent emission microwires can provide a potential platform to fabricate novel photonic and photoelectronic devices.
